# The Effect of Timing of Exercise and Eating on Postprandial Response in Adults: A Systematic Review

**DOI:** 10.3390/nu12010221

**Published:** 2020-01-15

**Authors:** Marah Aqeel, Anna Forster, Elizabeth A. Richards, Erin Hennessy, Bethany McGowan, Anindya Bhadra, Jiaqi Guo, Saul Gelfand, Edward Delp, Heather A. Eicher-Miller

**Affiliations:** 1 Department of Nutrition Science, Purdue University, West Lafayette, IN 47907, USA; aqeel@purdue.edu; 2 School of Nursing, Purdue University, West Lafayette, IN 47907, USA; aforste@purdue.edu (A.F.); earichar@purdue.edu (E.A.R.); 3 Friedman School of Nutrition Science and Policy, Tufts University, Boston, MA 02155, USA; erin.hennessy@tufts.edu; 4 Libraries and School of Information Studies, Purdue University, West Lafayette, IN 47907, USA; bmcgowa@purdue.edu; 5 Department of Statistics, Purdue University, West Lafayette, IN 47907, USA; bhadra@purdue.edu; 6 School of Electrical and Computer Engineering, Purdue University, West Lafayette, IN 47907, USA; guo498@purdue.edu (J.G.); gelfand@ecn.purdue.edu (S.G.); ace@ecn.purdue.edu (E.D.)

**Keywords:** timing, exercise, dietary intake, eating, postprandial response, glycemia, type 2 diabetes, healthy, overweight, obese

## Abstract

Type 2 diabetes is a major public health concern. Management of this condition has focused on behavior modification through diet and exercise interventions. A growing body of evidence has focused on temporality of dietary intake and exercise and potential effects on health. This review summarizes current literature that investigates the question “how does the timing of exercise relative to eating throughout the day effect postprandial response in adults?” Databases PubMed, Scopus, Cochrane Library, CINAHL, and SPORTDiscus were searched between March–May 2019. Experimental studies conducted in healthy adults (≥18 y) and those with type 2 diabetes were included. Full texts were examined by at least two independent reviewers. Twenty studies with a total of 352 participants met the inclusion criteria. The primary finding supports that exercise performed post-meal regardless of time of day had a beneficial impact on postprandial glycemia. There was insufficient evidence regarding whether timing of exercise performed pre- vs. post-meal or vice versa in a day is related to improved postprandial glycemic response due to inherent differences between studies. Future studies focusing on the investigation of timing and occurrence of meal intake and exercise throughout the day are needed to inform whether there is, and what is, an optimal time for these behaviors regarding long-term health outcomes.

## 1. Introduction

Type 2 diabetes (T2D) has increased globally and represents a major public health concern. An estimated 30.3 million people of all ages, 9.4% of the U.S. population, had diabetes in 2015 [1]. Underlying modifiable risk factors for T2D include behavioral and lifestyle habits such as dietary intake and physical activity patterns [2,3].

There is an abundance of research focused on the management of T2D, with most of the effort focusing on evaluating the effect of increased physical activity in combination with dietary interventions [2,4,5]. Specifically, increased exercise has been shown to aid in weight loss and maintenance [6], and improve insulin sensitivity [7] and glycemic control [8]; while a dietary pattern characterized by a high intake of fruits, vegetables, and whole grains and lower intake of processed products, meat, and sugar has been linked to a reduced risk of T2D [3].

A growing body of evidence has focused on temporality, or timing, and health behaviors to better understand whether or how time and behaviors like eating and exercising interact to influence health. For instance, the association between breakfast-skipping and eating later in the day, and adverse metabolic alterations [9,10] and increased risk of T2D, are prominent in this literature [11,12,13,14]. Fewer studies addressed timing of exercise with regard to weight and metabolic control, but preliminary data point to a possible association with health [15,16].

A considerable number of studies have examined the association between timing of exercise relative to a single meal or over a single day with health [17,18,19,20,21,22]. Joint consideration of the timing of these two behaviors is critical in the context of their potential synergistic relationship with long-term health. To our knowledge, there are no published reviews that focused on this investigation with emphasis on the time of day of these events. Therefore, the aim of this review is to summarize current literature that investigates the question; how does the timing of exercise relative to eating throughout the day effect postprandial response in adults?

## 2. Materials and Methods

### 2.1. Literature Search Strategy

**Figure 1:**
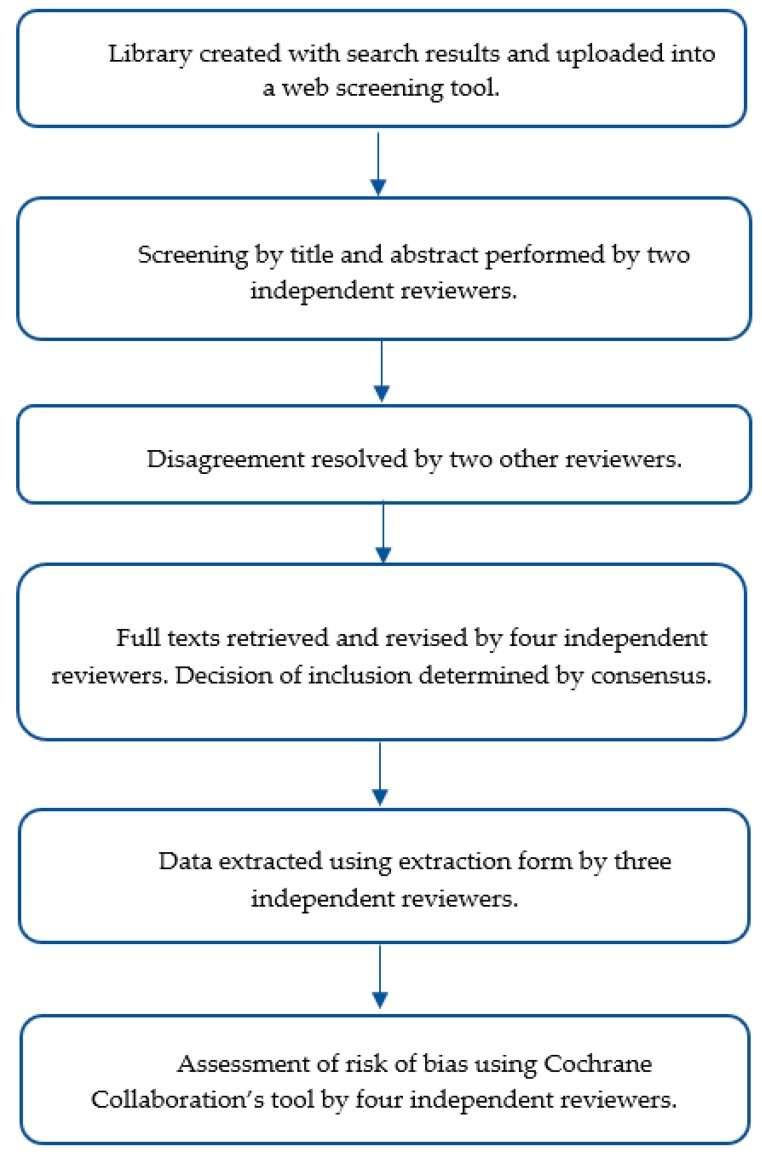
Flow chart representing the review process.

**Figure 2:**
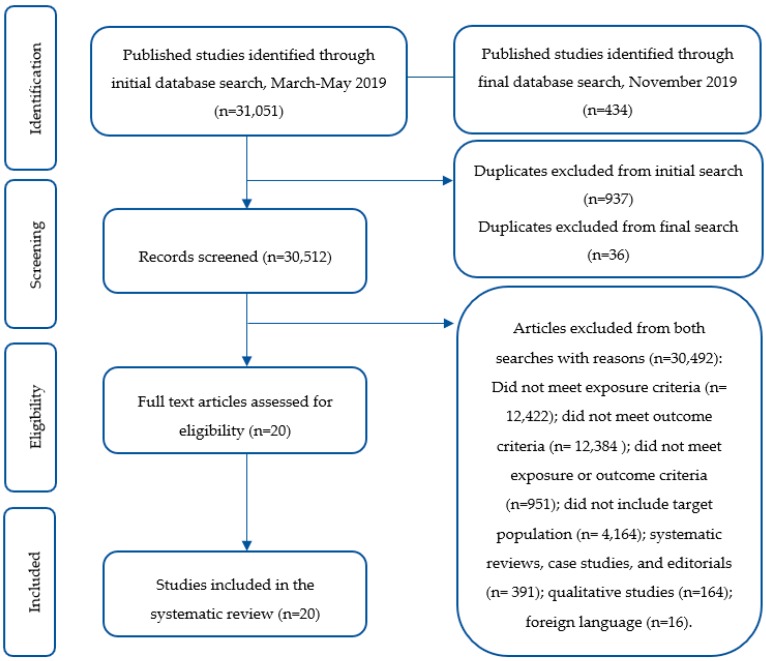
Flow chart representing selection of studies in the systematic review.

A health sciences librarian (B.M.) performed literature searches during March 2019 through May 2019 in the following databases: MEDLINE (via PubMed), SPORTDiscus, Scopus, Cochrane Library Database of Systematic Reviews, and CINAHL. A final search was executed in November 2019 to capture new publications. Searches designed for each database included controlled vocabulary terms (Medical Subject Headings), when applicable, and keywords (see Table S1 in the Supplementary Material). No filters were used during the search process. The literature was searched using combinations of terms including “meal timing” or “eating time” or “time of eating” or “eating patterns” or “eating behavior” or “ingestive behavior” or “exercise time” or “pre-prandial exercise” or “postprandial exercise”, and “obesity” and “overweight” and “Type 2 diabetes” (Figure 1 and Figure 2). PRISMA recommendations were followed and the study protocol was registered with Prospero (ID: CRD42019135459).

### 2.2. Types of Studies and Eligibility Criteria

Both experimental and observational studies were eligible for inclusion to be more inclusive of current evidence pertaining to the research question. Criteria specified healthy adults (≥18 years old) or individuals with overweight/obesity and/or T2D. Studies investigating the interaction of eating and exercising behaviors in the morning (i.e., exercise pre- or post-breakfast), evening (i.e., exercise pre- or post-dinner), or across an entire day (i.e., exercise pre- or-post several meals throughout the day) were eligible for inclusion. Studies involving children, women who were pregnant or lactating, or individuals with type 1 diabetes, gestational diabetes, or other chronic diseases were excluded from this review. 

### 2.3. Study Selection

Two reviewers independently screened articles retrieved from the search strategy by title and abstract simultaneously (M.A., A.F.) to determine inclusion. Articles with disagreement were allocated to two additional independent reviewers (H.A.E.-M., E.A.R.). Full texts were obtained for articles meeting inclusion criteria and were reviewed by four independent reviewers. Final study selection was determined by consensus. This systematic review included experimental trials reporting comparisons of different exercise timing interventions relative to meal consumption (e.g., exercise performed pre- or post-meal), which reported on postprandial (PP) glycemia.

### 2.4. Data Collection and Extraction

For studies meeting the inclusion criteria, three reviewers (M.A., A.F., E.H.) independently extracted data using an established data extraction form. The following information was extracted from the included studies; (i) study characteristics: Citation, publication year, setting, and purpose/objectives; (ii) inclusion and exclusion criteria; (iii) study design; (iv) sample characteristics: Sample location, size, demographic information, health status, and baseline anthropometric and metabolic variables; (v) description of the intervention; (vi) key results pertaining to the outcomes of interest; (vii) conclusions reported by the authors; (viii) funding sources and conflict of interest statement.

### 2.5. Study Quality and Assessment of Risk of Bias in Included Studies

The validity of each study was independently assessed by two reviewers (M.A., A.F.) using the Cochrane Collaboration’s tool for assessing risk of bias in randomized controlled trials (RCTs) [23]. Reviewers were not blinded to study authors or journal. The process involved critical assessment of several domains including selection bias (random sequence allocation and allocation concealment), performance bias (blinding of participants and personnel), detection bias (blinding of outcome assessment), attrition bias (incomplete outcome data), reporting bias (selective reporting), and other biases. Studies were further assessed by two additional reviewers (H.A.E.-M., E.A.R.), uncertainties were discussed, and consensus was reached in all cases.

### 2.6. Data Synthesis and Analysis

A narrative synthesis of the findings was conducted and structured around timing of exercise intervention relative to meal consumption throughout the day.

## 3. Results

### 3.1. Characteristics of Studies Included in the Review

Twenty studies met the inclusion criteria for this systematic review (Table 1). These studies included adults with T2D [17,18,20,21,22,24,25,26,27,28,29,30,31,32,33], as well as healthy [26,34,35,36] individuals and those with overweight/obesity and no reported comorbidities [19,37]. In terms of the interventions, included studies examined the effect of exercise performed relative to a meal on PP metabolic response. Eighteen studies included moderate-intensity aerobic exercise, three examined high-intensity training [17,18,22], and only one consisted of resistance exercise [29]. Furthermore, thirteen studies investigated exercise relative to breakfast/morning meal [17,19,20,21,22,26,27,28,31,32,35,36,37], four included dinner/evening meal [24,29,30,33], and three examined exercise performed at several time points throughout the day [18,25,34]. In this review, the results were organized by time of day of performing exercise relative to meal consumption to maintain a focus on the timing and order of these activities.

### 3.2. Risk of Bias Assessment

Most of the assessed studies provided insufficient information regarding randomization procedures (Table 2), whereas a few were non-randomized trials [21,36,37]. All of the studies were considered to be at low risk of bias for selective reporting because studies pre-specified their primary and secondary outcomes of interest. All of the studies reported the number of participants who completed the study but did not provide the total number of participants who initially enrolled. Additionally, blinding of participants and personnel was not feasible due to the nature of the interventions, thus this domain was deemed to be of low risk of bias; however, blinding of outcomes assessment was determined as unclear due to insufficient information for all included studies except Reynolds et al. [25] which was considered low risk due to blinding of their statistician to primary analysis. Incomplete outcome data were judged to be of low risk of bias in 13 of the included studies; the rest of the studies [19,22,27,28,29,30,36] were considered unclear due to insufficient reporting of attrition. 

### 3.3. Exercise Relative to Breakfast/Morning Meal Consumption

Studies that investigated the effect of time of exercise performance relative to a breakfast/morning meal on glycemic response were designed to examine PP glycemic response after acute intake of a standardized meal. These interventions included participants with T2D [17,20,21,22,26,27,28,31,32] and/or overweight/obese [19,37] as well as healthy individuals [35,36].

Studies in participants with T2D included an exercise intervention performed pre- [20,28] and post-meal [17,21,26,27,32], and pre- or post-breakfast [22,31]. Evidence regarding the glucose-lowering effect of exercise performed pre-breakfast was mixed. Oberlin et al. assessed the effect of pre-breakfast exercise on PP glycemic response to subsequent meals using continuous glucose monitors, for a two-day period under two conditions; no exercise (control), or 60 min of moderate-intensity exercise. The findings revealed that blood glucose concentration was significantly lower in the exercise condition compared to no exercise in the first 24 h period (*p* < 0.038). Additionally, compared to the control, exercise was associated with lower PP glucose area under the curve (AUC) across all meals on both days (*p* = 0.015). When comparing glycemic response to both conditions after each meal, lower PP glucose AUC was observed only after lunch (1:00 p.m.) on day 1 (*p* = 0.04) [28]. On the other hand, Ruegemer et al. examined the optimal timing of exercise in participants with insulin-dependent diabetes mellitus receiving intensive insulin therapy [20]. Participants were provided with standardized meals and were studied on four occasions including two at rest and two with 30 min exercise performed pre-breakfast (7 a.m.) or in the afternoon (4 p.m.). Findings revealed a significant increase in plasma glucose concentration in the pre-breakfast exercise condition compared to exercising in the afternoon or in the no exercise condition (*p* < 0.05), however, there was no difference in any of the groups 4 h after the meal. Thus, it remains unclear whether exercise performed pre-meal in the morning compared with no exercise is more advantageous for lowering PP glycemia in individuals with T2D.

Findings regarding the effect of morning post-meal exercise on glycemic response in participants with T2D were more consistent. Three crossover trials investigated the effect of moderate-intensity exercise performed post-breakfast on glycemic response using a similar methodological approach [21,27,32]. Participants were involved in two experimental conditions in a crossover design in which they were provided with a standardized meal and either exercised or remained sedentary afterward. Erickson et al. assessed whether post-meal exercise provided an additional glucose lowering effect, beyond medication alone, in patients using add-on hypoglycemic agents [21]. Glucose peak (drug only: 13.8 ± 3.7 mmol/L, drug/exercise: 9.9 ± 2.7 mmol/L) and glucose AUC (drug only: 500 ± 136 mmol/L, drug/exercise: 357 ± 89 mmol/L) were significantly lower during the time of the exercise bout (*p* = 0.02 and *p* = 0.03, respectively); moreover, compared to the control, average 2 h incremental AUC (iAUC) during the breakfast PP period was significantly lower on the exercise day (*p* = 0.047). Two other studies found a beneficial effect of post-meal exercise on PP glucose levels in participants with T2D [27,32] and revealed that this effect persisted at least for the duration of the next meal (*p* < 0.05) [32]. A finding that supported this evidence while evaluating the effect of varying duration of high intensity exercise revealed that exercise for 30 min post-meal significantly reduced blood glucose concentration to a greater extent compared to 60 and 90 min of post-meal exercise or no exercise [17]. Nelson et al. further confirmed these findings in a randomized controlled trial that characterized the metabolic response to moderate-intensity exercise performed post-meal in healthy individuals (controls) and those with insulin-dependent diabetes mellitus [26]. Participants were provided with a standardized breakfast after which they either rested for 3 h or exercised for 45 min. Compared to no exercise, PP glycemic response was significantly lower in the exercise condition between 45–75 min and 65–95 min in controls and participants with insulin-dependent diabetes mellitus, respectively (both *p* < 0.05). Therefore, compared with no exercise, post-meal exercise performed in the morning is more effective at attenuating PP glycemic response in participants with T2D.

Two studies investigated the effect of pre- and post-meal exercise performed in the morning on PP response among individuals with T2D and reported inconsistent findings [22,31]. Poirier et al. compared changes in blood glucose levels in response to 1 h of moderate-intensity exercise performed pre-breakfast or 2 h after consumption of a standardized breakfast meal [31]. Compared to baseline, blood glucose concentration was significantly lower in both conditions (*p* = 0.003 and *p* < 0.001, respectively); however, the reduction in the post-meal condition was sustained during the recovery period while it returned to pre-exercise levels in the pre-breakfast exercise condition. Conversely, Terada et al. compared pre- vs. post-breakfast walking (60-min of continuous moderate intensity exercise or intervals of 1 min high/3 min lower intensity) to a no exercise control condition [22]. Compared to post-meal exercise, pre-meal exercise was more effective at reducing PP glycemic increments (*p* < 0.05). Moreover, high-intensity interval exercise lowered mean nocturnal and fasting glucose to a larger extent compared to moderate-intensity continuous exercise (both *p* < 0.05). When comparing all exercise conditions to control, pre-meal high-intensity exercise performed in the morning lowered mean amplitude of glycemic excursion and total post-meal iAUC (*p* < 0.05). Hence, it is unknown whether morning pre- vs. post-meal exercise is more effective at lowering PP glycemia in participants with T2D.

Two studies conducted in individuals with obesity (diabetes prone) and those with overweight assessed glycemic response to moderate-intensity exercise performed post-breakfast [37] and pre- or post-breakfast [19], respectively. Lunde et al. showed that compared to a no exercise condition, post-meal walking attenuated the glycemic response to a carbohydrate-rich meal with improved outcomes with longer walking duration (40 min vs. 20 min). Contrary to these reports, Farah et al. assigned participants to three experimental conditions including no exercise (control) and exercise pre- or post-breakfast. There was no significant difference in glucose response over an 8.5 h observation period between conditions; however, compared to the control, both pre- and post-meal exercise lowered insulin response (by 19% and 24% in pre-meal and post-meal, respectively, both *p* < 0.01), while only pre-breakfast exercise was associated with lower PP triglyceride (*p* = 0.025). Among those with overweight and obesity, morning exercise was shown to be effective at improving PP metabolic response with no clear benefit based on timing of exercise relative to meal consumption.

Studies among healthy participants included an exercise intervention performed post-breakfast [35,36]. One intervention assessed the effect of post-breakfast walking on glycemic response (Three conditions: No exercise, 15 or 40 min walking) [35]. Findings revealed that post-meal exercise performed in the morning attenuated the glycemic response to a carbohydrate-rich meal and this effect was enhanced with prolonged walking duration. Similarly, another study revealed lower peak blood glucose value in the exercise condition compared to no exercise irrespective of age and training condition [36]. Amongst healthy participants, compared with no exercise, post-meal exercise performed in the morning is more effective at attenuating PP glycemic response.

In summary, studies reporting the effect of exercise performed relative to a morning meal on glycemic response were mostly drawn from randomized crossover trials that either examined PP response to a breakfast/morning meal or monitored response to successive meals. Consistently, results showed that exercise performed in the morning post-meal had an advantageous effect on PP glycemia in participants with T2D, overweight/obese, and healthy subjects. However, results were less consistent in regards to the effect of pre-meal exercise performed in the morning on glycemia in participants with T2D. Additionally, three studies, conducted in individuals with T2D [22,31] and those who were overweight [19], directly assessed the effect of exercise performed pre- vs. post-breakfast on glycemia and resulted in inconclusive findings.

### 3.4. Exercise Relative to Dinner/Evening Meal Consumption

Evidence regarding the effect of exercise performed relative to a dinner/evening meal on glycemic response has resulted from randomized crossover trials in participants with T2D [24,29,30,33]. Li et al. evaluated the effect of post-dinner exercise on glycemic response using continuous glucose monitors [24]. Participants consumed a standardized diet and were randomized to two experimental conditions including a no exercise group (control) or a 20 min post-dinner exercise group. Significant declines in 2 h PP glucose spike (*p* = 0.04), peak glucose (*p* = 0.02), and mean glucose (*p* = 0.04) levels were reported under the exercise condition compared to the control. Moreover, compared to the control, 12 h standard deviation of blood glucose and the coefficient of variation of glucose were both significantly lower in the exercise condition (both *p* < 0.009), while mean amplitude of glycemic excursion was not significantly different. Supporting this evidence, two other studies examined the effect of resistance [29] or aerobic [30] exercise performed pre- or post-dinner on cardiovascular disease risk factors [29] and glycemic control [30] with a similar methodological approach. Participants were randomized to three experimental conditions including no exercise (control), pre-, or post-dinner exercise and were provided with standardized meals on experimental days. Researchers reported similar findings of improved markers of cardiometabolic control (significant lower triglyceride and improved insulin clearance) [29] and lower blood glucose values [30] with both exercise types performed post-dinner. Interestingly, a study by Rees et al. investigating the effect of walking pre-dinner on 24 h glycemic outcomes revealed no difference in most of the examined glycemic variables including 24 h glucose, fasting glucose, PP glucose, and glucose variability in an exercise condition compared to the control condition [33]. In summary, evidence regarding the effect of exercise relative to consumption of a dinner/evening meal on glycemic response was drawn from studies conducted in participants with T2D. Findings revealed that exercise in the evening has an advantageous effect on PP glycemic response with a potential superior effect in post-dinner exercise compared to pre-dinner exercise.

### 3.5. Divided Exercise Bouts vs. Conventional Continuous Sessions Performed Pre- or Post-Meals Consumed Throughout the Day

Studies that investigated the effect of continuous vs. divided exercise bouts performed pre- or post-meal throughout the day on glycemic response were conducted in participants with T2D [18,25] and healthy individuals [34]. Reynolds et al. randomized participants to two experimental conditions, each lasting for 2 weeks, and included walking for 30 min at any time of day (conventional) or walking for 10 min post main meals [25]. PP glucose iAUC was 12% lower in the post-meal compared to conventional condition; additionally, 3 h mean blood glucose was significantly reduced after the evening meal in the post-meal vs. conventional condition (*p* = 0.034). Nevertheless, it is important to note a significant difference in overall physical activity (counts/minute) between the two exercise conditions (*p* = 0.006) explained by reduced sedentary time and increased walking duration in the post-meal condition. A study that supported this evidence of reduced PP glucose concentration with brief bouts of exercise compared to a single bout also added that this effect persisted for the subsequent 24 h with pre-meal exercise [18]. Supporting these results in healthy participants, Manohar et al. quantified the effect of low-intensity exercise on glycemic variability in individuals consuming a standardized diet for three days at fixed times [34]. Participants walked for 5–6 h each day and were assessed using a physical activity monitor; in random order, one meal per day was followed by inactivity, and the other two meals were followed by walking. The PP glucose iAUC was significantly lower in meals followed by walking compared to meals followed with inactivity (*p* = 0.022). In summary, these studies showed that exercise has a beneficial effect on glycemic response in healthy participants and those with T2D. Furthermore, brief bouts of exercise pre- or post-meals performed throughout the day could be more beneficial compared to one continuous exercise bout in participants with T2D.

## 4. Discussion

This review sought to investigate current literature focused on the temporality of health behaviors to better understand whether, or how, time and activities of eating and exercising interact to influence health. Most research that integrated these concepts included randomized crossover trials conducted in participants with T2D. Studies mainly examined the effect of exercise performed relative to a morning or evening meal on PP glycemic response, given the importance of this component in T2D management [21]. Twenty crossover studies with a total of 352 participants were included in this review. The primary findings were: (1) Exercise performed post-meal regardless of time of day had a beneficial impact on PP glycemia including lower plasma glucose concentration and glucose AUC; and, (2) there was insufficient evidence regarding whether the timing of exercise performed (e.g., pre- vs. post-meal) throughout the day is related to improved PP glycemic response.

Exercise performed in the morning post-meal in participants with T2D, healthy, and obese individuals was consistently linked to acute attenuation in PP glycemia compared to sedentary controls (2–3 h after ingestion of a meal) [17,21,26,27,32,35,36,37]. These findings align with observations by Haxhi et al. in individuals with T2D and healthy participants [38]. The blunting effect of PP exercise on peak blood glucose level has been well established [31,36,39]. These results may be explained by an elevation in endogenous insulin stimulated by food intake [30] as well as muscular contraction which co-act to enhance skeletal muscle glucose uptake independent of insulin [40]. Only three studies assessed the effect of exercise performed post-meal in the evening [24] or post several meals throughout the day [25,34] and reported an advantageous effect on glycemic response. 

Fewer studies examined the association between pre-meal exercise performed in the morning [20,28] and the evening [33] and metabolic response in participants with T2D. Studies conducted in the morning resulted in inconsistent findings, with one trial reporting elevated blood glucose in response to pre-breakfast exercise [20]; while another revealed a decrease in mean 24 h glucose concentration in exercise conditions compared to control, although, the improvement from exercise was observed in the second meal (~4.5 h post-exercise) but not in the first meal (~30 min post-exercise) [28]. The large heterogeneity of these two studies is an important consideration. Specifically, studies differed in mean age of recruited participants (30.0 vs. 60.1 year), health condition (IDDM vs. T2D), duration of exercise (30 vs. 60 min), and duration of PP blood sampling (up to 3 vs. 24 h) in [20,28], respectively. Only one study investigated the effect of walking pre-meal in the evening and reported no difference in most examined glycemic outcomes in the exercise condition compared to no exercise [33]. A possible explanation for this finding is related to the timing of exercise which was performed 3–5 h post-lunch and 20 min pre-dinner. The limited evidence from studies examining pre-meal exercise and glycemic response may be further elucidated by future randomized controlled trials overcoming these limitations. 

Relating to this investigation, five studies compared the effect of pre- vs. post-meal exercise performed in the morning [19,22,31] or evening [29,30] on glycemic response. Studies that examined the effect of exercise performed pre- or post-meal in the morning resulted in inconclusive findings. One crossover trial including healthy participants [19] and another involving high-intensity interval training [22] reported a larger attenuation in PP glycemia with exercise performed pre- compared to post-breakfast. Conversely, a study including participants with T2D revealed significantly lower blood glucose levels when moderate-intensity exercise was completed 2 h post- rather than pre-breakfast [31]. The discrepancy in these findings could be related to differences in the type of exercise and health status of the study population (healthy vs. T2D). Moreover, studies differed in frequency and duration of blood sampling, specifically, post-exercise glucose concentration was measured at 30–60 min, 15 min, or continuous intervals (using continuous glucose monitor) up to 7, 1.5, or 24 h in [19,22,31], respectively. Differences also existed in timing of the exercise bout relative to meal consumption; exercise was performed in the fasted state and at 30, 60, or 120 min post-meal in the morning in [19,22,31], respectively. On the other hand, two crossover trials examined the effect of exercise performed in the evening pre- or post-meal in participants with T2D and resulted in more consistent findings. Colberg et al. reported lower blood glucose levels with the completion of 20 min of walking, starting 15–20 min post-dinner, compared to pre-dinner or no walking [30]. Contrastingly, both pre- and post-dinner resistance exercise similarly improved blood glucose AUC subsequent to dinner irrespective of timing in Heden et al.; however, lower PP triglyceride was reported with post-dinner exercise suggesting a potential superior benefit on metabolic response [29]. The inherent differences in study design including time of day of exercise and eating (morning vs. evening), type of exercise (aerobic vs. resistance), and intensity and modality (moderate continuous vs. high intensity interval training) preclude conclusions regarding optimal exercise–meal timing throughout the day for management of glycemic response. In addition, the above findings resulted from short-term studies that typically included 10–13 participants observed in a controlled setting with standardized meals provided; therefore, further longer-term studies preferably simulating “real life” settings are needed to demonstrate whether modulating time of exercise relative to meals translates into an overall improvement in glycemic control. 

Of note, there was insufficient evidence in the reviewed studies regarding whether and how the time of day of eating and exercising interact to impact health outcomes. Only three studies investigated the effect of exercise performed pre- or post- several meals consumed during the day on PP response [18,25,34]; however, their main goal was to examine whether continuous vs. divided bouts of exercise could differentially effect PP glycemia. Additionally, although current findings indicate that post-meal exercise regardless of time of day has an advantageous impact on PP response, available evidence is limited by a focus on blocks of time during the day, specifically the morning period. Future studies that emphasize time of day of these behaviors, i.e., including both exercise and eating conditions in the morning and evening or across an entire day, are warranted to inform whether time of day of these behaviors is indeed related to clinically meaningful effects on health. 

Most of the studies included in this review involved mild- or moderate-intensity exercise and showed an advantageous effect on PP glycemic response. Moderate-intensity exercise is the most commonly recommended strategy for individuals with impaired glucose tolerance or T2D [41]. Self-paced exercise for 20 min in the evening resulted in lower plasma glucose levels compared to a no exercise condition in one study [30]. However, high-intensity interval training has gained recent attention as a time-efficient strategy for metabolic disease management [17,42]. Compared to no exercise, a single bout of high-intensity interval training performed in the morning was associated with reduced same-day PP glucose AUC [22]. Additionally, performing brief bouts of exercise (six 1 min uphill walking intervals at 90% HRmax) before main meals throughout the day was more effective at reducing PP hyperglycemia when compared with a single bout of pre-dinner moderate-intensity walking (30 min, ~60% HRmax) [18]. Limited evidence exists examining the effect of high-intensity interval training on PP glycemic response; thus, this training modality is under-represented in this review; nevertheless, available findings suggest that engaging in any physical activity mode and intensity throughout the day lowers PP glycemic response in individuals with T2D.

One major strength of this review included the selection of recently published studies investigating the interaction between eating and exercising behaviors while emphasizing the sequence of these behaviors throughout the day and their potential effect on health. Additionally, most of the studies included both male and female participants with approximately 50% of the study sample being female. However, collectively, the studies were not representative of a certain population nor group, which limits generalizability. Major limitations pertain to the crossover nature of the interventions, with lack of a control group in a few studies [18,25,31]. Moreover, three of the included trials were non-randomized [21,36,37] and most others were unclear in terms of their randomization procedures. Additionally, included studies varied with regards to time of day of eating and exercise, duration of assessment, type of exercise, and timing between exercise and meals, and were short-term in nature; nonetheless, findings were similar in showing an advantageous effect of post-meal exercise in lowering PP hyperglycemia regardless of time of day and pointing to a potential benefit of modulating exercise timing relative to meal consumption for optimizing metabolic control. Of note, most of the included studies examined the effect of exercise performed relative to a morning meal on health status indicators, while there was inadequate evidence emphasizing the time of day of both eating and exercising, and their potential associations with health. Considering that the distribution of eating and exercising throughout the day has a repetitious pattern, timing of these activities, in isolation and relative to each other, throughout the day could have an important link to health. Future studies focusing on the investigation of timing and occurrence of these behaviors across an entire day are needed to inform whether the development of time-specific recommendations is relevant to improved long-term health outcomes including T2D.

## 5. Conclusions

In conclusion, the findings of this systematic review show a beneficial effect of post-meal exercise on improved PP glycemic response regardless of time of day in healthy individuals and participants with overweight/obesity and/or T2D. However, findings were less clear regarding optimal exercise–meal timing for enhanced glycemic response due to inherent differences between studies. Moreover, studies pertaining to this investigation have mostly resulted from randomized crossover trials with the provision of standardized meals; thus, more studies simulating “real life” settings are needed to elucidate how timing of eating and exercising throughout the day interact to influence long-term health outcomes.

## Figures and Tables

**Table 1:** Summary of studies included in the systematic review.

Citation	Study Length	*n* (M: F)	Age (Years) Mean ± SD	Disease Condition	Intervention/ Comparison	Treatment Effects (↑, ↓, ↔)	Assessment Duration	Glycemic Response	
								PP Plasma Glucose	Glucose AUC
	**Exercise relative to breakfast/morning meal consumption**								
Caron et al. [32]	2 days	8 (5:3)	24.25 ± NA	IDDM	Control (no exercise) vs. exercise post-breakfast	↓ meal glycemic excursions	NA	Intervention day: Rise in glycemia was attenuated and remained constant during exercise at 190 ± 33 mg/dL; glycemia decreased to 163 ± 30 mg/dL just before lunch; after the second meal, all values from 300 min onward were significantly lower with exercise vs. control	Not reported
Erickson et al. [21]	2 days	8 (5:3)	60 ± 10.7	Obesity/T2D	Control day (no exercise) vs. exercise post-breakfast	↓ glucose	2 h	Exercise post-breakfast: Significantly lower PG peak and decreasing glucose over time	Significant difference in average on 2 h iAUC
Huang et al. [17]	4 days	26 (12:14)	53.8 ± 8.6	OW/T2D	No exercise (control) vs. exercise post-breakfast (EX30) vs. exercise 60 min post-breakfast (EX60) vs. exercise 90 min post-breakfast (EX90)	↓ PPG	NA	Compared to control, declines in PG immediately post-exercise were larger in EX30, EX60, and EX90; capillary glucose decreased significantly after exercise in EX30, EX60, and EX90	Not reported
Nelson et al. [26]	2 days	16 (10:6)	Control group: 33.3 ± 2 Experimental: 25.7 ± 2	IDDM	Control (no exercise) vs. post-breakfast exercise	↓ glycemia	0, 30, 75, 95, 135, 180 min.	Post-breakfast exercise: Significant ↓ in glycemia	Not reported
Oberlin et al. [28]	4 days	9 (4:5)	60.1 ± 1	Obesity/T2D	Control (no exercise) vs. pre-breakfast exercise	↓ 24 h average blood glucose	24 h avg glucose, 4 h glucose AUC, 2 h PPG	Pre-breakfast exercise significantly lowered avg. PG concentration during first 24 h period compared to control (5.98 vs. 6.62 mmol/L)	Main effect of exercise to lower PPG-AUC across all 6 meals compared to control
Poirier et al. [31]	2 days	10 (10:0)	54 ± 5	Sedentary/T2D	Exercise pre-meal vs. post-breakfast exercise	↓ plasma glucose (post-meal exercise)	2 h	Mean decrease in PG concentration was 4.8 ± 1.9 mmol/L (60 ± 14% of baseline) vs. 1.0 ± 0.8 mmol/L (91 ± 6% of baseline) in post-meal vs. pre-meal exercise, respectively. Significantly lower PG level in post-meal vs. pre-meal condition (7.6 vs. 10.0 mmol/L)	Not reported
Rasmussen et al. [27]	2 days	7 (7:0)	29 ± 4	IDDM	Control (no exercise) vs. post-early meal (11:30 a.m.) exercise	↓ blood glucose	−30, 0, 15, 30, 45, 60, 90, 120, 180 min	Post-meal exercise: Significantly lower PG response areas by 34% vs. control (638 vs. 492 mmol/L × 180 min)	Not reported
Ruegemer et al. [20]	4 days	6 (3:3)	30 ± 4	IDDM	Control (no exercise) vs. pre-breakfast exercise vs. pre- meal (4:00 p.m.) exercise	↑ plasma glucose (exercise pre-breakfast)	−20, −10, 0, 15, 30, 45, 60, 75, 90, 120, 180, 240, 300, 360 min	Significant hyperglycemic response to morning exercise; no difference between groups 4 h after meals	Not reported
Terada et al. [22]	5 days	10 (8:2)	60 ± 6	Obesity/T2D	Control (no exercise) vs. pre-breakfast HIIE vs. post-breakfast HIIE vs. pre-breakfast MICE vs. post-breakfast MICE	↓ PPG	24 h mean interstitial glucose concentration, 1 h mean PPG	Compared to post-meal exercise, pre-meal condition significantly attenuated PP glycemic increments	Comparing all exercise conditions to control, pre-meal HIIE significantly lowered total post-meal iAUC
Farah et al. [19]	3 days	10 (10:0)	28.1 ± 10.7	OW	Control (no exercise) vs. pre-breakfast vs. post-breakfast exercise	↔ PPG	7 h PPG	No difference in glycemic response between conditions	Not reported
Lunde et al. [37]	3 days	11 (0:11)	44 ± NA	Obesity/diabetes prone	Control (no exercise) vs. post-breakfast 20 min. walk vs. post-breakfast 40 min walk	↓ PPG	2 h PPG	PPG and PG peak value significantly decreased with increasing duration of slow post-breakfast walking	2 h glucose iAUC decreased with increasing duration of slow post-meal walking
Høstmark et al. [36]	2 days	39 (0:39)	Trained young: 22.5 ± 0.5 Trained middle-age: 49.2 ± 1.3 Sedentary young: 24.1 ± 0.7 Sedentary middle-age: 59.2 ± 1.7	Sedentary and trained	Control vs. exercise post- breakfast	↓ peak glucose value ↓ blood glucose	NA	Exercise post-breakfast: Peak PG was lower than control	Not reported
Nygaard et al. [35]	3 days	13 (0:13)	Not listed	Healthy	Control (no exercise) vs. post-breakfast 15 min. walk vs. post-breakfast 40 min walk	↓ blood glucose	15, 22.5, 30, 37.5, 45, 55, 65, 75, 90, 105, 120 min	Compared to control, peak PG value was 0.8 mmol/L lower (significant) in post-breakfast 40 min walk condition	Significant main effect of walking time on 2 h iAUC; participants with the largest 2 h PG iAUC on the control day demonstrated the greatest reduction in PPG response when walking 40 min post-breakfast
**Exercise relative to dinner/ evening meal consumption**									
Colberg et al. [30]	3 days	12 (6:0)	61.47 ± 2.7	Obesity/T2D	Control day (no exercise) vs. exercise pre-dinner vs. exercise post-dinner	↓ plasma glucose	4 h	Exercise post-dinner: Significantly lower PG levels at the end of exercise compared to at the same time point when participants had exercised pre-dinner	Total glucose AUC over 4 h was not significantly different among trials
Heden et al. [29]	3 days	13 (5:8)	48.5 ± 11.9	Obesity/T2D	No resistance exercise (control) vs. pre-dinner resistance exercise vs. post-dinner resistance exercise	↓ glucose iAUC (exercise pre-meal)	NA	Not reported	Significant reduction in glucose iAUC by ~18% and 30% in pre- and post-dinner exercise, respectively
Li et al. [24]	2 days	29 (22:7)	51 ± 11.2	T2D	Control (no exercise) vs. post-dinner exercise	↓ PP hyperglycemia	2 h PPG	Post-dinner exercise vs. control: Significant lowering in 2 h PPG spike (1.9 ± 1.3 vs. 2.7 ± 1.4 mmol/L), 2 h PP peak glucose (9.3 ± 1.6 vs. 10.3 ± 2.3 mmol/L), and 2 h PP mean glucose levels (8.2 ± 1.3 vs. 8.9 ± 2.0 mmol/L)	Post-dinner exercise: Glucose tAUC 1 h after exercise was significantly lower than control (493.9 ± 84.0 vs. 559.3 ± 130.5 mmol/L × 60 min)
Rees et al. [33]	1 week	73 (33:40)	63.5 ± 9.1	Obesity/T2D	Control (no exercise) vs. pre-dinner walking	↓ blood glucose	24 h glucose, 2 h PPG	Exercise had no effect on PPG or 24 h glucose variability; significant reduction in PG concentration during walking in exercise condition vs. control (−1.56 mmol/L)	Not reported
**Divided bouts vs. conventional continuous exercise performed pre- or post-meals consumed throughout the day**									
Francois et al. [18]	3 days	9 (7:2)	48 ± 6	Obesity/insulin resistant T2D	Control (continuous exercise pre-dinner) vs. exercise snacking pre-mean meals (ES) vs. composite exercise snacking pre- main meals (CES)	↓ mean PPG (post- dinner and breakfast)	3 h PPG and mean PPG, 24 h glucose concentration	ES significantly attenuated mean 3 h PPG concentrations following breakfast (0.4 ± 1.0 mmol/L) and 24 h mean PG concentrations by 0.7± 0.6 mmol/L relative to baseline	Not reported
Manohar et al. [34]	3 days	12 (5:7)	37.7 ± 13.7	Healthy	Control (no exercise) vs. post-meal exercise	↓ PPG excursions	NA	Baseline CGM PG concentration lower with post-meal exercise vs. control (5.61 mmol/L vs. 5.58 mmol/L); peak CGM PG concentration lower with post-meal exercise vs. control (8.25 mmol/L and 11.99 mmol/L)	Post-meal exercise: iAUC was estimated to be significantly lower than control (4.5 mmol/L/270 min vs. 9.6 mmol/L/270 min), respectively
Reynolds et al. [25]	2 weeks	41 (26:15)	60 ± 9.9	Obesity/T2D	30 min walk at any time of day vs. 10 min walk post 3 main meals	↓ PPG	3 h	Significantly lower 3 h mean PG levels following evening meal with post-meal walking compared to conventional condition (−0.50 mmol/L)	Glucose iAUC was 12% lower in the post-meal compared to conventional condition

Abbreviations: M (male); F (female); Avg (average); PP (postprandial); PG (plasma glucose); PPG (postprandial glucose); AUC (area under the curve); tAUC (total AUC); iAUC (incremental AUC); HIIE (high intensity interval exercise); MICE (moderate intensity continuous exercise); IDDM (insulin-dependent diabetes mellitus); OW (overweight); CGM (continuous glucose monitoring); ↑ (increase); ↓ (decrease); ↔ (no change).

**Table 2:** Risk of bias assessment of included studies.

Author [ref]	Random Sequence Generation	Allocation Concealment	Selective Reporting	Blinding		Incomplete Outcome Data	Other Bias
				Participants/personnel	Outcomes assessment		
Caron et al. [32]	U	L	L	L	U	L	L
Colberg et al. [30]	U	L	L	L	U	U	L
Erickson et al. [21]	H	L	L	L	U	L	L
Farah et al. [19]	L	L	L	L	U	U	L
Francois et al. [18]	U	L	L	L	U	L	L
Heden et al. [29]	U	L	L	L	U	U	L
Høstmark et al. [36]	H	L	L	L	U	U	L
Huang et al. [17]	U	L	L	L	U	L	L
Li et al. [24]	U	L	L	L	U	L	L
Lunde et al. [37]	H	L	L	L	U	L	L
Manohar et al. [34]	U	L	L	L	U	L	M
Nelson et al. [26]	H	L	L	L	U	L	M
Nygaard et al. [35]	U	L	L	L	U	L	L
Oberlin et al. [28]	U	L	L	L	U	U	L
Poirier et al. [31]	U	L	L	L	U	L	L
Rasmussen et al. [27]	U	L	L	L	U	U	L
Rees et al. [33]	L	L	L	L	U	L	L
Reynolds et al. [25]	L	L	L	L	L	L	L
Ruegemer et al. [20]	U	L	L	L	U	L	L
Terada et al. [22]	L	L	L	L	U	U	L

Abbreviations: H (high risk of bias); M (moderate risk of bias); L (low risk of bias); U (unclear risk of bias).

## Supplementary Materials

The following are available online at https://www.mdpi.com/2072-6643/12/1/221/s1, Table S1: Search Strategy.
