# Correction: Aqeel, M.; et al. “The Effect of Timing of Exercise and Eating on Postprandial Response in Adults: A Systematic Review”. *Nutrients*, 2020, *12*, 221

**DOI:** 10.3390/nu12051263

**Published:** 2020-04-29

**Authors:** Marah Aqeel, Anna Forster, Elizabeth A. Richards, Erin Hennessy, Bethany McGowan, Anindya Bhadra, Jiaqi Guo, Saul Gelfand, Edward Delp, Heather A. Eicher-Miller

**Affiliations:** 1 Department of Nutrition Science, Purdue University, West Lafayette, IN 47907, USA; aqeel@purdue.edu; 2 School of Nursing, Purdue University, West Lafayette, IN 47907, USA; aforste@purdue.edu (A.F.); earichar@purdue.edu (E.A.R.); 3 Friedman School of Nutrition Science and Policy, Tufts University, Boston, MA 02155, USA; erin.hennessy@tufts.edu; 4 Libraries and School of Information Studies, Purdue University, West Lafayette, IN 47907, USA; bmcgowa@purdue.edu; 5 Department of Statistics, Purdue University, West Lafayette, IN 47907, USA; bhadra@purdue.edu; 6 School of Electrical and Computer Engineering, Purdue University, West Lafayette, IN 47907, USA; guo498@purdue.edu (J.G.); gelfand@ecn.purdue.edu (S.G.); ace@ecn.purdue.edu (E.D.)

We would like to submit the following corrections to our recently published paper [1] after discovering the inclusion of three studies in our review that did not meet the inclusion criteria we had specified in our methods: healthy adults (≥18 years old) or individuals with overweight/obesity and/or type 2 diabetes (T2D). Specifically, all three studies included participants with type 1 diabetes (T1D) which was part of our exclusion criteria as described in our manuscript. The reason for the original inclusion of these three studies stems from confusion in the older terminology “insulin-dependent diabetes” that was eventually eliminated and replaced with “T1D” since the term insulin-dependent diabetes mellitus “has been confusing and has frequently resulted in classifying the patient based on treatment rather than etiology” as stated by the Expert Committee on the Diagnosis and Classification of Diabetes Mellitus report [2]. We feel the correction explained herein is an example of this confusion and the purpose for updating this terminology. Furthermore, this correction could serve as a reminder to readers who do not work in this subject extensively to note the ambiguity surrounding the old terminology. Removal of these studies does not change the overall or specific results or conclusions, since these three studies contributed to the largest section of studies addressing “exercise relative to breakfast/morning meal consumption,” and resulted in similar findings. Therefore, revisions did not impact the hypothesis, results, and conclusions of the review.

**Abstract:** In the original paper, the total number of included studies was *n* = 20 and is *n* = 17 in the revised version. Also, in the original paper, the total number of participants was *n* = 352, while it is *n* = 332 in the revised paper.

**1. Materials and Methods**: In the original paper, Figure 2 specified the inclusion of 20 studies, while the revised paper specifies *n* = 17 included studies.

**2. Results**: In the original paper, **page 4, paragraph 1**, we stated “**Twenty** studies met the inclusion criteria for this systematic review (Table 1) **Eighteen** studies included moderate-intensity aerobic exercise, three examined high-intensity. Furthermore, **thirteen** studies investigated exercise relative to breakfast/morning meal [17,19–22,26–28,31,32,35–37], four included dinner/evening meal [24,29,30,33], and three examined exercise performed at several time points throughout the day [18,25,34].”

The revised version should read as follows “**Seventeen** studies met the inclusion criteria for this systematic review (Table 1) **Fifteen** studies included moderate-intensity aerobic exercise, three examined high-intensity Furthermore, **ten** studies investigated exercise relative to breakfast/morning meal [17,19–21,25,28,31–34], four included dinner/evening meal [23,26,27,29], and three examined exercise performed at several time points throughout the day [18,24,30].”

In the original paper, **Table 1:** summary of studies included in the systematic review, **section:** exercise relative to breakfast/morning meal consumption, included studies by Caron et al. (reference 32), Rasmussen et al. (reference 27), and Ruegemer et al. (reference 20). In the revised paper, we have removed all three studies from this section. Also, in the original version, the study by Nelson et al. (reference 26) specified insulin-dependent diabetes mellitus (IDDM) as the condition with *n* = 16; the revised version specifies healthy individuals with *n* = 7 (This study included healthy subjects and those with IDDM; sample persons and results pertaining to subjects with IDDM were removed).

Additionally, the original paper included all three studies (Caron et al., Rasmussen et al., and Ruegemer et al.) in **Table 2:** risk of bias assessment of included studies, which were removed in the revised paper.

In the original paper, in the section “exercise relative to breakfast/morning meal consumption” **pages 11–12, paragraph 2**, the sentence “Evidence regarding the glucose lowering effect of exercise performed pre-breakfast was mixed” has been modified in the revised version as “Evidence regarding the glucose lowering effect of exercise performed pre-meal was limited.”

In the original paper, in the section “exercise relative to breakfast/morning meal consumption” **pages 11–12, paragraph 2**, the sentences “On the other hand, Ruegemer et al. examined the optimal timing of exercise in participants with insulin-dependent diabetes mellitus receiving intensive insulin therapy [20]. Participants were provided with standardized meals and were studied on four occasions including two at rest and two with 30 min exercise performed pre-breakfast (7 a.m.) or in the afternoon (4 p.m.). Findings revealed a significant increase in plasma glucose concentration in the pre-breakfast exercise condition compared to exercising in the afternoon or in the no exercise condition (*p* < 0.05), however, there was no difference in any of the groups 4 h after the meal.” have been removed from the revised paper.

In the original paper, in the section “exercise relative to breakfast/morning meal consumption”, **page 12, paragraph 3**, the sentence “Three crossover trials investigated the effect of moderate-intensity exercise performed post-breakfast on glycemic response using a similar methodological approach [21,27,32].” has been removed from the revised version.

In the original paper, in the section “exercise relative to breakfast/morning meal consumption”, **page 12, paragraph 3**, the statement “Two other studies found a beneficial effect of post-meal exercise on PP glucose levels in participants with T2D [27,32] and revealed that this effect persisted at least for the duration of the next meal (p < 0.05) [32]” has been removed from the revised paper.

In the original paper, **page 12, paragraph 3**, we stated “Nelson et al. further confirmed these findings in a randomized controlled trial that characterized the metabolic response to moderate-intensity exercise performed post-meal in healthy individuals (controls) and those with insulin-dependent diabetes mellitus [26]. Participants were provided with a standardized breakfast after which they either rested for 3 h or exercised for 45 min. Compared to no exercise, PP glycemic response was significantly lower in the exercise condition between 45–75 min and 65–95 min in controls and participants with insulin-dependent diabetes mellitus, respectively (both *p* < 0.05).”

In the revised paper, we edited this paragraph to read “Nelson et al. further confirmed these findings in a randomized controlled trial that characterized the metabolic response to moderate-intensity exercise performed post-meal in healthy individuals [33]. Participants were provided with a standardized breakfast after which they either rested for 3 h or exercised for 45 min. Compared to no exercise, PP glycemic response was significantly lower in the exercise condition between 45–75 min (*p* < 0.05).” We additionally moved this paragraph to the section pertaining to studies on exercise performed post-breakfast among healthy participants.

In the original paper, **page 13, paragraph 7**, the sentence “However, results were less consistent in regards to the effect of pre-meal exercise performed in the morning on glycemia in participants with T2D” has been revised to “However, results were limited in regards to the effect of pre-meal exercise performed in the morning on glycemia in participants with T2D”.

**3. Discussion**: In the original paper, **paragraph 1, page14**, we specified *n* = 352 as the total number of participants and twenty as the total number of studies, while the revised version includes *n* = 332 and seventeen studies as the total number of included studies.

In the original paper, **page 14, paragraph 3,** we stated “Studies conducted in the morning resulted in inconsistent findings, with one trial reporting elevated blood glucose in response to pre-breakfast exercise [20]; while another revealed a decrease in mean 24 h glucose concentration in exercise conditions compared to control, although, the improvement from exercise was observed in the second (~4.5 h post-exercise) but not in the first meal (~30 min post-exercise) [28].” This sentence was revised to read as follows “One study was conducted in the morning and revealed a decrease in mean 24 h glucose concentration in exercise conditions compared to control, although, the improvement from exercise was observed in the second meal (~4.5 h post-exercise) but not in the first meal (~30 min post-exercise) [25].”

In the original paper, **pages 14–15, paragraph 3**, we stated “The large heterogeneity of these two studies is an important consideration. Specifically, studies differed in mean age of recruited participants (30.0 vs. 60.1 year), health condition (IDDM vs. T2D), duration of exercise (30 vs. 60 min), and duration of PP blood sampling (up to 3 vs. 24 h) in [20,28], respectively.” These two sentences were removed from the revised paper.

In the original paper, **page 15, paragraph 3,** we stated “Only one study investigated the effect of walking pre-meal in the evening and reported no difference in most examined glycemic outcomes in the exercise condition compared to no exercise [33].” This sentence was revised to read as follows “Additionally, Rees et al. investigated the effect of walking pre-meal in the evening and reported no difference in most examined glycemic outcomes in the exercise condition compared to no exercise [29].”

**4. Conclusions**: No changes were made to this section.

The authors apologize to the readers for any inconvenience caused by this amendment. This amendment does not affect the results or conclusion of the manuscript in any way. The original manuscript will remain online on the article webpage with a reference to this correction.
