# Supplementary material for: The Effect of Timing of Exercise and Eating on Postprandial Response in Adults: A Systematic Review
Source: Nutrients. 2020 Jan 15;12(1):221. doi: 10.3390/nu12010221 (PMC7019516; doi:10.3390/nu12010221)
Supplement: Supplementary file 1 [file nutrients-12-00221-s001.pdf]

Table S1. Search Strategy:

Initial search strategy executed March – May 2019		
**Database**	**Search Strategy**	**Number of results**
**Medline PubMed**	("Adult"[Mesh]) AND (Meal Timing[tiab] OR eating time[tiab] OR ingestive behavior[tiab] OR time of eating[tiab] OR eating patterns[tiab] OR feeding behavior[MESH] OR "Exercise"[Mesh] OR exercise time[tiab] OR pre-prandial exercise[tiab] OR postprandial exercise[tiab]) AND Humans AND (obesity[tiab] OR overweight[tiab] OR obesity[mesh] OR diabetes[tiab] OR Diabetes Mellitus[mesh])	26,401
**SPORTDiscus**	(adult AND (eating AND behavior OR feeding AND behavior OR meal AND timing OR eating AND patterns OR exercise AND time) AND humans AND (obesity OR overweight OR diabetes))	113
**CINAHL**	Adult AND (eating behavior OR feeding behavior OR ingestive behavior OR meal timing OR eating patterns OR eating time OR exercise time or pre-prandial exercise or post-postprandial exercise or exercise OR time of eating) AND humans AND (obesity or overweight or diabetes or diabetes mellitus)	4,206
**Cochrane Library Database of Systematic Reviews**	Adult AND (eating behavior OR feeding behavior OR ingestive behavior OR meal timing OR eating patterns OR eating time OR exercise time OR pre-prandial exercise OR post-prandial exercise OR exercise OR time of eating) AND humans AND (obesity or overweight or diabetes or diabetes mellitus)	42
**Scopus**	(adult AND (eating AND behavior OR feeding AND behavior OR meal AND timing OR eating AND patterns OR exercise AND time) AND humans AND (obesity OR overweight OR diabetes))	277
**Google Scholar**	(adult AND (eating AND behavior OR feeding AND behavior OR meal AND timing OR eating AND patterns OR exercise AND time) AND humans AND (obesity OR overweight OR diabetes))	12

Total results= 31,051
Number of duplicates removed= 937
Total number of studies screened: 30,114
Studies excluded for not meeting eligibility criteria: 30,095
Studies included for full text assessment: 19
Studies included in review: 19

Table S1. Search Strategy Cont.

Final search executed on November 17, 2019 for period May 21 - November 17		
**Database**	**Search Strategy**	**Number of results**
**Medline PubMed**	("Adult"[Mesh]) AND (Meal Timing[tiab] OR eating time[tiab] OR ingestive behavior[tiab] OR time of eating[tiab] OR eating patterns[tiab] OR feeding behavior[MESH] OR "Exercise"[Mesh] OR exercise time[tiab] OR pre-prandial exercise[tiab] OR postprandial exercise[tiab]) AND Humans AND (obesity[tiab] OR overweight[tiab] OR obesity[mesh] OR diabetes[tiab] OR Diabetes Mellitus[mesh])	135
**SPORTDiscus**	(adult AND (eating AND behavior OR feeding AND behavior OR meal AND timing OR eating AND patterns OR exercise AND time) AND humans AND (obesity OR overweight OR diabetes))	19
**CINAHL**	Adult AND (eating behavior OR feeding behavior OR ingestive behavior OR meal timing OR eating patterns OR eating time OR exercise time or pre-prandial exercise or post-postprandial exercise or exercise OR time of eating) AND humans AND (obesity or overweight or diabetes or diabetes mellitus)	226
**Cochrane Library Database of Systematic Reviews**	Adult AND (eating behavior OR feeding behavior OR ingestive behavior OR meal timing OR eating patterns OR eating time OR exercise time OR pre-prandial exercise OR post-prandial exercise OR exercise OR time of eating) AND humans AND (obesity or overweight or diabetes or diabetes mellitus)	2
**Scopus**	(adult AND (eating AND behavior OR feeding AND behavior OR meal AND timing OR eating AND patterns OR exercise AND time) AND humans AND (obesity OR overweight OR diabetes))	50
**Google Scholar**	(adult AND (eating AND behavior OR feeding AND behavior OR meal AND timing OR eating AND patterns OR exercise AND time) AND humans AND (obesity OR overweight OR diabetes))	2

Total number of studies screened = 434
Number of duplicates= 36
Total number of studies screened: 398
Studies excluded for not meeting eligibility criteria: 397
Studies included for full text assessment: 1
Studies included in review: 1
